# Key Stakeholder Priorities for the Review and Update of the Australian Guide to Diagnosis of Fetal Alcohol Spectrum Disorder: A Qualitative Descriptive Study

**DOI:** 10.3390/ijerph19105823

**Published:** 2022-05-10

**Authors:** Nicole Hayes, Lisa K. Akison, Sarah Goldsbury, Nicole Hewlett, Elizabeth J. Elliott, Amy Finlay-Jones, Dianne C. Shanley, Kerryn Bagley, Andi Crawford, Haydn Till, Alison Crichton, Rowena Friend, Karen M. Moritz, Raewyn Mutch, Sophie Harrington, Andrew Webster, Natasha Reid

**Affiliations:** 1 Child Health Research Centre, The University of Queensland, Brisbane, QLD 4072, Australia; l.akison@uq.edu.au (L.K.A.); k.moritz1@uq.edu.au (K.M.M.); n.reid1@uq.edu.au (N.R.); 2 School of Biomedical Sciences, The University of Queensland, Brisbane, QLD 4072, Australia; 3 Māori Indigenous Health Innovation, University of Otago Christchurch, Christchurch 8013, New Zealand; sarahgoldsburypsychology@gmail.com; 4 Centre for Healthcare Transformation, Queensland University of Technology, Brisbane, QLD 4059, Australia; nicole.hewlett@qut.edu.au; 5 Wellbeing and Preventable Chronic Diseases Division, Menzies School of Health Research, Darwin, NT 0811, Australia; 6 National Organisation for Fetal Alcohol Spectrum Disorder Australia, Adelaide, SA 5000, Australia; sophie.harrington@nofasd.org.au; 7 Faculty of Medicine and Health, Specialty of Child and Adolescent Health, The University of Sydney, Sydney, NSW 2006, Australia; elizabeth.elliott@health.nsw.gov.au; 8 The Sydney Children’s Hospitals Network, Sydney, NSW 2145, Australia; 9 Telethon Kids Institute, Perth, WA 6009, Australia; amy.finlay-jones@telethonkids.org.au (A.F.-J.); raewyn.mutch@health.wa.gov.au (R.M.); 10 School of Population Health, Curtin University, Perth, WA 6102, Australia; 11 Menzies Health Institute of Queensland, Griffith University, Gold Coast, QLD 4222, Australia; d.shanley@griffith.edu.au; 12 School of Applied Psychology, Griffith University, Gold Coast, QLD 4222, Australia; 13 La Trobe Rural Health School, La Trobe University, Bendigo, VIC 3552, Australia; k.bagley@latrobe.edu.au; 14 Living with Disability Research Centre, La Trobe University, Melbourne, VIC 3083, Australia; 15 Department of Psychological Medicine, Faculty of Medical and Health Science, The University of Auckland, Auckland 1023, New Zealand; andi@crawford.ac.nz; 16 Te Ara Manapou, Parenting and Pregnancy Service, Hawke’s Bay District Health Board, Hastings 4120, New Zealand; 17 Child Development Service, Gold Coast Hospital and Health Service, Southport, QLD 4215, Australia; haydn.till@health.qld.gov.au; 18 Victoria Fetal Alcohol Service, Monash Children’s Hospital, Clayton, VIC 3163, Australia; alison.crichton@monashhealth.org; 19 Department of Paediatrics, Monash University, Clayton, VIC 3163, Australia; 20 Patches Assessment Service, Berrimbah, NT 0828, Australia; rowena.friend@patches.com.au; 21 Child and Adolescent Health Service, Department of Health, Perth Children’s Hospital, Perth, WA 6009, Australia; 22 Faculty of Health Sciences, Curtin Medical School, Curtin University, Perth, WA 6102, Australia; 23 School of Medicine, Dentistry and Health Sciences, University of Western Australia, Perth, WA 6009, Australia; 24 Harvard Program for Refugee Trauma, Continuing Medical Education, Harvard Medical School, Boston, MA 02115, USA; 25 West Moreton Health, Ipswich, QLD 4305, Australia; andrew.webster@health.qld.gov.au

**Keywords:** fetal alcohol spectrum disorder, prenatal alcohol exposure, clinical guidelines, assessment and diagnosis

## Abstract

Since the 2016 release of the Australian Guide to the Diagnosis of Fetal Alcohol Spectrum Disorder (FASD), considerable progress has been made in the identification and diagnosis of the disorder. As part of a larger process to review and update the Guide, the aim of this study was to identify review priorities from a broad range of stakeholders involved in the assessment and diagnosis of FASD. Sixty-two stakeholders, including healthcare practitioners, researchers, other specialists, individuals with cultural expertise, lived experience and consumer representatives completed an online survey asking them to describe up to five priorities for the review of the Australian Guide to the Diagnosis of FASD. A total of 267 priorities were described. Content analysis of responses revealed priority areas relating to diagnostic criteria (*n* = 82, 30.7%), guideline content (*n* = 91, 34.1%), guideline dissemination (*n* = 15, 5.6%) and guideline implementation (*n* = 63, 23.6%). Other considerations included prevention and screening of FASD (*n* = 16, 6%). Engaging stakeholders in setting priorities will ensure the revised Australian Guide can be as relevant and meaningful as possible for the primary end-users and that it meets the needs of individuals with lived experience who will be most affected by the diagnosis.

## 1. Introduction

Fetal alcohol spectrum disorder (FASD) is a complex neurodevelopmental condition that arises from prenatal alcohol exposure and is characterised by a broad spectrum of lifelong physical, cognitive, and behavioural difficulties [1]. Individuals with FASD often have multiple comorbidities and without timely intervention, can experience adverse outcomes and secondary conditions, including disengagement from school, mental health and substance abuse disorders, limited ability to live and work independently and involvement with the legal system [2,3]. The global prevalence of FASD among young people in the general population is estimated to be approximately 7.7 per 1000 population [4], making FASD one of the most common preventable causes of developmental disability. There are substantial economic costs associated with FASD due to the lifelong support that can be required and research from the United States, Canada, Sweden and New Zealand estimates a mean annual cost for children with FASD to be USD $22,810 [5] and an estimated lifetime cost of at least USD $2 million per individual [6].

In recognition of the need for improved diagnosis of FASD in Australia, the Australian Government funded the development and dissemination of The Australian Guide to the Diagnosis of FASD in 2016 [7]. The Guide aims to provide a framework to give healthcare practitioners the confidence to consider a diagnosis of FASD, the knowledge to make the diagnosis and the information they need to manage or refer an individual and family. The Guide was based on a literature review and consultation with stakeholders, was an adaptation of the Canadian National Guidelines [1] and incorporated the lip-philtrum guide from the University of Washington’s 4-Digit Diagnostic Code [8]. A key recommendation in the Australian Guide is the adoption of FASD as a diagnostic term, with two subcategories: FASD with three sentinel facial features and FASD with less than three sentinel facial features [9]. Diagnostic criteria include evidence of prenatal alcohol exposure, severe impairment in at least three out of 10 specified neurodevelopmental domains and presence of sentinel facial features (see Table 1). A multidisciplinary diagnostic assessment process, involving paediatricians, psychologists, occupational and speech therapists is recommended to ensure comprehensive physical and developmental assessments, as are laboratory tests including genetics. The Guide also provides recommendations and resources for discussing the diagnosis and developing a management plan. To ensure the Guide reflects current knowledge and best practice in the evolving field of FASD, a priority objective identified in the 2018–2028 National Action Plan for FASD in Australia [10] was to review the Australian Guide to the Diagnosis of FASD and ensure alignment with international best practice and adoption of emerging evidence-based practices.

Clinical practice guidelines are systematically developed statements that aim to facilitate good clinical practice by assisting healthcare practitioners in making decisions about diagnosis and/or treatment for specific conditions [11]. Along with considering the empirical evidence, an important element of guideline development is the inclusion and consideration of stakeholder input. Stakeholders include those who might be expected or intended to apply, implement, or be otherwise directly affected by the guideline recommendations, including people with direct and indirect lived experience of the condition in question [11,12]. Increasingly, the quality of clinical practice guidelines is appraised and evaluated based on their involvement and consideration of stakeholder perspectives [13]. A guideline developed with input from stakeholders is more likely to be relevant to the needs of its expected users, enhancing its legitimacy and improving dissemination and implementation [14,15].

For the diagnosis of FASD, key professional stakeholders include healthcare practitioners in primary care, specialist medical fields (e.g., paediatricians, psychiatrists, geneticist) and allied health disciplines (e.g., psychology, occupational therapy, physiotherapy, speech and language pathology and social work) who are involved in the identification, assessment, diagnosis, and support of individuals with FASD. Other stakeholders include professionals such as educators and justice personnel who work with individuals with FASD; individuals with cultural knowledge and expertise; academic researchers studying the sequalae of prenatal alcohol exposure; and importantly, people with lived experience of FASD (e.g., parents, caregivers, and individuals with FASD). Since the release of the Guide in 2016, significant progress in Australia has been made towards consistent diagnostic practices and improving diagnostic capacity of FASD [16]. However, understanding facilitators and barriers to current guideline use and identifying opportunities for improvement will strengthen revisions and maximise guideline uptake and impact in policy and practice. Although several Australian studies have examined community priorities with regard to FASD research [17] and health professionals’ familiarity with and use of existing FASD diagnostic guidelines [18], no other study has examined priorities specific to FASD clinical practice guidelines in Australia that considers a broad range of stakeholder perspectives.

In 2020, a national consortium led by 14 steering committee members across 12 university and professional organisations received funding from the Australian Government to review and update the existing Australian Guide to the Diagnosis of FASD, published in 2016. The aim of the current study was to obtain stakeholder perspectives on FASD assessment and diagnosis in Australia to inform the revision of the guide. Specifically, we wanted to determine the priorities of healthcare professionals, researchers, other specialists, and people living with/caring for individuals with FASD who will ultimately use and be affected by the revised guide. By engaging key stakeholders in setting priorities, the revised Australian Guide can be as relevant and meaningful as possible for the primary end-users (i.e., health professionals), and benefit people living with FASD. Stakeholder engagement is part of the larger guideline review process, which involves systematically reviewing the international literature and other international guidelines.

## 2. Materials and Methods

This research utilized a qualitative online survey design to examine key stakeholder priorities for the review of the Australian FASD Diagnostic Guide.

### 2.1. Participants and Recruitment

Key stakeholders including healthcare practitioners, researchers, other specialists, individuals with cultural expertise, lived experience and consumer representatives were invited to take part in Advisory Groups to inform the review and update of the Australian FASD Diagnostic Guide. Key stakeholders were recruited via relevant Australian professional associations (e.g., Australian and New Zealand FASD Clinical Network, Royal Australian College of General Practitioners, Neurodevelopmental and Paediatric Society of Australia, Australian Psychological Society); FASD-related consumer organisations (e.g., National Organisation for Fetal Alcohol Spectrum Disorder Australia, The Russell Family Fetal Alcohol Association); and relevant First Nations professional bodies (e.g., The First People’s Disability Network of Australia, National Aboriginal Community Controlled Health Organisation). Known individuals with expertise in the FASD field were also contacted and invited to participate in an advisory group. Twenty-eight professional associations and organisations, and 56 expert individuals (i.e., including health, justice, education, and child protection) were contacted via email and provided with an Advisory Group expression of interest (EOI) form to distribute to their members and colleagues via newsletters, noticeboards and/or member distribution lists. Information regarding reasons to return/not return an EOI form was not collected. Key stakeholders who returned an expression of interest form were then contacted individually via email and provided with details regarding the online survey.

### 2.2. Measures

The email provided a web link to a de-identified online survey managed using REDCap electronic data capture [19], hosted at The University of Queensland. The survey was developed by two authors (N.R. and N.Ha) in consultation with all other authors. The survey contained seven questions, took approximately 10 min to complete and was able to be completed in a web browser on any device (e.g., phone, tablet, or computer). A copy of the survey is presented in the Supplementary Materials. The Save and Return option in REDCap was not enabled. According to the REDCap Status Dashboard, there were no incomplete surveys. The survey was open for completion from March to May 2021.

#### 2.2.1. Demographics

Six questions sought information about participants’ background demographics including gender, state/territory of residence, and advisory capacity (healthcare practitioner, researcher, other specialist, or individuals with lived experience/representative). For professionals, information was collected on the primary discipline area, years of experience in primary discipline area and years of experience working with individuals with FASD.

#### 2.2.2. Priority Areas for the Review of the Australian FASD Assessment and Diagnostic Guide

One question asked participants to list and describe up to five priorities they felt were important for the review of the Australian FASD Assessment and Diagnostic Guide. Participants provided written responses in five separate textboxes with no restriction on the maximum number of characters.

### 2.3. Ethical Aspects

This study was approved by the Children’s Health Queensland Hospital and Health Service Human Research Ethics Committee (HREC/20/QCHQ/69561) and The University of Queensland Human Research Ethics Committee (2021/HE000360). The online survey was completed anonymously, and survey responses were confidential.

### 2.4. Data Analysis

Responses to the open-ended question were analysed using content analysis to identify common priority areas and determine the frequency of these priority areas. Content analysis is a technique for studying responses to open-ended questions by counting and coding written words and text into categories and patterns [20,21]. Words and phrases within each individual priority response were identified and coded. Codes were then categorised to identify common elements across all priorities described by the participants. Each individual priority received only one code. To facilitate an evidence-based approach to the development of implementation strategies, suggested priorities related to implementation were categorised according to a conceptual framework for guideline implementability [22] across eight domains including: (1) adaptability (availability of versions of the guide for different uses or purposes); (2) usability (content is organised to enhance ease of use); (3) validity (evidence is summarised and can be easily reviewed); (4) applicability (contextual clinical information provided to interpret and apply recommendations); (5) communicability (information provided to support discussions with patients); (6) accommodation (costs, resources and training needs identified); (7) implementation (identified barriers to use, and promotional strategies described); and (8) evaluation (audit and monitoring measures).

Identification and coding of words and phrases were undertaken by one author (N.R.) and verified by a second author (N.Ha.). Both authors have experience in qualitative research methods and undertake clinical research in the FASD field, as well as on broader child development and wellbeing. N.R. is also a clinician with experience in the assessment and diagnosis of FASD.

## 3. Results

Ninety-four stakeholders returned the expression of interest form and received an email invitation to the online survey. Sixty-two stakeholders completed the online survey (response rate = 70%). Sample demographics are presented in Table 2. Most participants were female (*n* = 53) and were from Queensland (*n* = 20), Western Australia (*n* = 13), New South Wales (*n* = 10), Victoria (*n* = 8), Northern Territory (*n* = 7) and South Australia (*n* = 3). Fifty-five stakeholders were professionals, and seven stakeholders were individuals with lived experience/representatives. Primary professional discipline areas included psychology/neuropsychology (*n* = 16), allied health (speech pathology, occupational therapy, physiotherapy; *n* = 9), perinatal, paediatric, and public health research (*n* = 9), paediatrics (*n* = 7), and criminal/youth justice system (*n* = 5). Other primary discipline areas included disability support, social work, psychiatry, nursing, primary care addiction, genetics, and cultural expertise. Years of experience in the primary discipline area ranged from 2 to 44 years (M = 17.05 years, SD = 9.87). Years of experience working with individuals with FASD ranged from 0 to 30 years (M = 9.84 years, SD = 7.78).

### 3.1. Summary of Key Stakeholder Priorities

**Figure 1:**
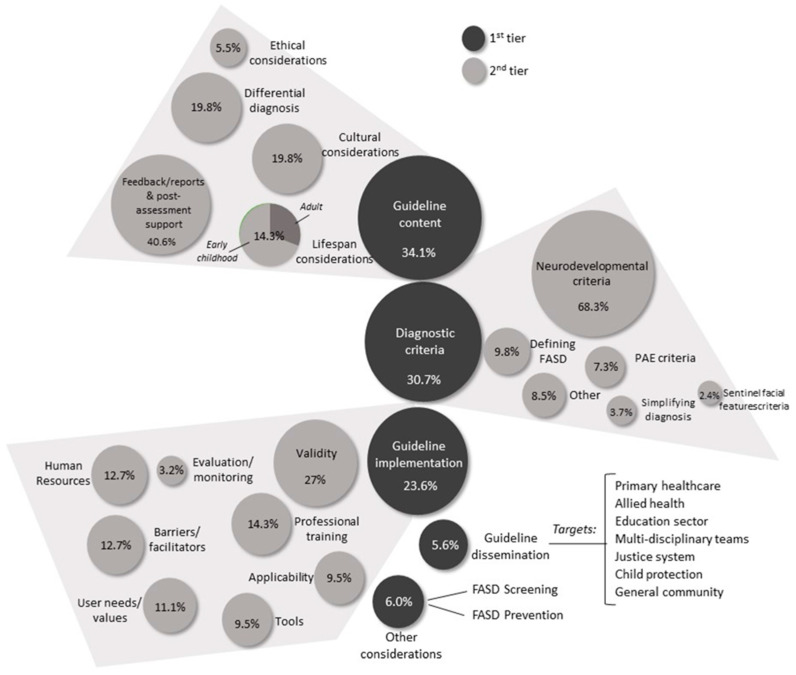
Stakeholder priority areas for the review of the Australian Guide to the Diagnosis of FASD.

Across all participants, a total of 267 priorities were described, with a median of 5 priorities described per participant (range 1–5). Of this, professionals contributed 233 priorities (87.3%) and individuals with lived experience and representatives contributed 34 priorities (12.7%). Key priority areas are presented in Figure 1. A breakdown of suggested priorities within key content areas along with supporting participant quotes is presented in Table 3. Priorities related to features of the diagnostic criteria (*n* = 82, 30.7%), guideline content (*n* = 91, 34.1%), dissemination (*n* = 15, 5.6%), and implementation (*n* = 63, 23.6%). Other considerations include prevention and screening of FASD (*n* = 16, 6%).

#### 3.1.1. Diagnostic Criteria

Priorities relating to the neurodevelopmental criteria were most common (*n* = 56, 68.3%). A priority was to review the conceptualisation and consideration of neurodevelopmental domains of the FASD diagnostic criteria, particularly in the context of acknowledging the “overlap of symptoms and how impairment in three of the 10 domains may not reflect widespread brain injury” (i.e., if the three domains a person presents with impairments in are inter-related/overlapping domains). Specific issues for review in the neurodevelopmental domains included considering the inclusion of sensory processing, considering the possible exclusion of academic achievement, clarifying and justifying the affect regulation domain, and considering the separation of adaptive and social communication skills domain. There was also an identified need to review the definition of ‘impairment’ and the currently recommended cut-off scores (i.e., ≤2 standard deviations below the mean). Other priorities included updating the suggested assessment tools, particularly the indirect (self- or parent-report) assessments; considering the requirement for functional assessments to be completed as part of the diagnostic process; aligning the neurodevelopmental criteria with other diagnostic guidelines such as Developmental Language Disorder under the language domain and Developmental Coordination Disorder under the motor domain.

Almost 10% (*n* = 8) of priorities related to diagnostic criteria involved defining FASD. Participants wanted clarification on the definition of FASD, particularly regarding the causative role of prenatal alcohol exposure. Participants also acknowledged that there are individuals living with mild to moderate impairments following prenatal alcohol exposure and wanted consideration of how the ‘spectrum’ of impairments (and related thresholds) could be subsumed under the umbrella of FASD. The remaining priorities within the diagnostic criteria topic related to the clarity of the prenatal alcohol exposure criteria and review of the inclusion of a threshold level of exposure (*n* = 6, 7.3%), review of sentinel facial features criteria (*n* = 2, 2.4%) in the context of appropriate normative data for Australian populations and simplifying the assessment and diagnosis procedures (*n* = 3, 3.7%).

#### 3.1.2. Guideline Content

For this theme, priorities related to post-assessment feedback, reports and follow-up support were most common (*n* = 37, 41.7%). This was also the most salient priority for individuals with lived experience and representatives (*n* = 12, 35.3% of total lived experience stakeholder priorities). Participants described the desire for recommendations to be provided on the process of providing the diagnosis and relevant assessment feedback to individuals and families and the need for consistent and minimum standards for diagnostic reports. Participants also described a desire for guidance on developing management plans and recommendations for post-assessment support and resources. A key area highlighted by participants was the need for increased support and coordination for individuals and families across contexts, including diversion in justice and culturally appropriate community-led supports.

Another key priority related to the guideline content features was cultural considerations (*n* = 18, 19.8%). Participants described the importance of ensuring the guide is culturally sensitive, safe, and inclusive for populations that experience access barriers, including First Nations peoples and individuals and families with non-English speaking backgrounds. Participants highlighted the need to consider alternative culturally appropriate assessment tools and clinical decision-making processes.

The topic of formulation, differential diagnosis, and co-occurring conditions (*n* = 18, 19.8%) was also identified as a key priority for the Guide. Participants described a need to provide guidance around considering differential diagnoses in the assessment process to determine the likelihood of alcohol-related impairments and identifying other causes or conditions that may present with similar features (e.g., other genetic conditions, other neurodevelopmental disorders). There was also a strong desire for the Guide to consider the impact of other co-occurring conditions such as sleep disturbances and physical health conditions and the role of external influences on neurodevelopment including trauma and poverty identified in assessments.

Almost 15% (*n* = 13) of recommended priorities within this theme related to lifespan considerations. Participants identified the importance of early diagnosis and the need to review how the assessments are completed in young children (i.e., <6 years), as well as the need for clearer guidelines for assessment and diagnosis in adults.

A final priority within this theme was ethical considerations (*n* = 5, 5.5%). Participants raised concerns regarding the implications of an FASD diagnosis, given the potential for stigma, blame and shame for individuals and communities. Thus, participants reported a desire for the guide to include a discussion on the implications of diagnosis and misdiagnosis and highlighted the need for advice to be provided to ensure informed consent for referral and assessment.

#### 3.1.3. Guideline Dissemination

The most common priority for this theme was the desire for widespread dissemination of the revised Guide beyond the health system to include education, justice, child protection and the general community (*n* = 12, 80%). This wide dissemination was described as a strategy to facilitate awareness, recognition and understanding of FASD within the wider professional and community settings, and was an important priority identified by individuals with lived experience and representatives (*n* = 5, 14.7% of total lived experience stakeholder priorities).

Another identified dissemination priority related to the need for targeted dissemination to multidisciplinary health teams including state-wide child development units (*n* = 2, 13.3%). It was also identified that there was a need to have a specific dissemination strategy for primary health professionals (*n* = 1, 6.7%) through established educational pathways and professional associations.

#### 3.1.4. Guideline Implementation

The most common implementation priority related to ensuring the validity of the Guide (*n* = 17, 27%). This included the need for an up-to-date review with supporting documentation and considering harmonisation with international diagnostic approaches to ensure alignment with international best practices. Another implementation priority was applicability (*n* = 6, 9.5%), and included ensuring the guide is clinically focused, presented in a user-friendly manner, and written in non-judgmental and patient-centred language.

Participants identified several priorities related to guideline accommodations (*n* = 24, 9%), including considering user needs and values related to the incorporation of lived experiences and end-users’ perceptions; human resources related to multi-disciplinary assessments and recommended alternatives to expand access when multi-disciplinary assessments are not feasible; and recommendations for professional training.

Other implementation priorities addressed barriers and facilitators (*n* = 8, 12.7%), tools (*n* = 6, 9.5%) and evaluation (*n* = 2, 3.2%). This included accessing prenatal care information, pathways of care for children with known prenatal alcohol exposure, the need for a register of FASD diagnostic clinics and practitioners, and monitoring of the implementation of the guide.

#### 3.1.5. Other Considerations

Other priority considerations related to prevention opportunities (*n* = 8, 50%) and the role of screening (*n* = 8, 50%). Although slightly outside the scope of most guidelines, participants identified the importance of a multi-level approach to prevention of FASD, including broad community awareness, engaging with women of childbearing years and their partners as part of discussions of health and lifestyle, and importantly, the need for honest, culturally safe, and non-judgmental prenatal care regarding alcohol use for women who are pregnant. Participants also identified the desire for recommendations regarding FASD screening and referral pathways when there is suspected prenatal alcohol exposure history or identified FASD concern when children present for developmental assessments.

## 4. Discussion

The aim of the present study was to gather key stakeholder perspectives, including those of healthcare practitioners, researchers, other specialists, individuals with cultural expertise, people with lived experience and consumer representatives regarding key priorities for the review and update of the current Australian Guide to the Diagnosis of FASD. Four main priority areas were identified that related to (1) diagnostic criteria, (2) guideline content, (3) guideline dissemination, and (4) guideline implementation. Other considerations identified included FASD screening and prevention. This is the first study to formally document the priorities of key stakeholders from a broad range of disciplines and backgrounds regarding clinical practice guidelines in Australia for the assessment and diagnosis of FASD. The findings demonstrate the value of stakeholder feedback to inform revisions to the Australian Guide and clinical practice guidelines more generally.

### 4.1. Stakeholder Priorities and Implications for Guideline Revisions

#### 4.1.1. Diagnostic Criteria

Priorities relating to the diagnostic criteria focused on the neurodevelopmental domains assessed as part of the FASD diagnostic process. There was a strong desire from stakeholders for a review of the conceptualisation of the 10 neurodevelopmental domains identified in the current guide, with particular emphasis on overlap across domains. Whilst the current Australian Guide presents the 10 domains separately, clinical judgement is encouraged as “domains should not be assessed as though they were separate entities” [7] (p. 18). It is notable that there are differences in how other international FASD diagnostic guidelines conceptualise the neurodevelopmental criteria. For example, the proposed Diagnostic and Statistical Manual of Mental Disorders (DSM) criteria for Neurobehavioural Disorder associated with Prenatal Alcohol Exposure (ND-PAE) provides three overarching areas (i.e., neurocognitive, self-regulation and adaptive behaviour), under which multiple individual domains are included [23]. This higher-level conceptualisation could assist with the challenges healthcare practitioners reported in the current study regarding the overlap when assessing the 10 specified neurodevelopmental domains.

The definition of ‘severe impairment’ and clinical cut-offs on the neurodevelopmental domains was also identified as a key priority for review. In the current Australian Guide, scores at or below two standard deviations (3rd percentile) of the mean are considered ‘severe impairment’ as this is “the usual standard for defining a severe level of impairment” [7] (p. 16). Internationally, there is also a discrepancy between FASD diagnostic guidelines regarding the clinical cut-offs used for diagnosis (see [24,25] for a review). For example, the Revised Institute of Medicine (IOM) Guidelines [26] utilise a cut-off of 1.5 standard deviations below the mean and the University of Washington’s 4-Digit Diagnostic Code [8] includes different diagnostic outcomes based on the level of impairment (e.g., those with moderate impairments receive a diagnosis of neurobehavioural disorder—alcohol exposed). The discrepancies in the FASD field are in line with the absence of a clear definition in the broader neuropsychology field, as there are no universally agreed criteria for impairment [27,28,29]. Cut-offs of 1, 1.5 and 2 standard deviations below normative scores have been used to define impairment [28]. Importantly, Guilmette and colleagues [28] note that defining impairment based purely on test scores that deviate from the norm is an “inappropriate concrete approach that considers each specific test score as having inherent clinical meaning, without considering the overall test result profile and the particular examinee’s life context. Such an approach is not viewed as an acceptable method of arriving at clinical conclusions” (p. 440).

Stakeholders highlighted the importance of considering and assessing functional impacts as part of the diagnostic process. This was also noted by Guilmette and colleagues [28] as an important consideration in applying a definition of impairment in an individual case. Specifically, they noted that average test scores do not preclude the determination of functional limitations. Consideration of context is required, and environmental and task demands, as well as supports that ameliorate or mitigate capacity, should be considered. One strategy for addressing this in future guidelines might be to recommend that assessment results are linked to observed or reported behaviour challenges. When brain and behaviour are linked explicitly, helpful management strategies are often easier to suggest, resulting in more user-friendly, meaningful reports.

Stakeholders prioritised the need to clarify the causal role of prenatal alcohol exposure in the definition of FASD, and how prenatal alcohol exposure is related to observed impairments. Consistent with the revised Canadian Guidelines [1], in the current Australian Guide [7], FASD is used as a diagnostic term with two sub-categories that relate to the presence or absence of sentinel facial features. The presence of facial features directly implicates prenatal alcohol exposure, and the use of ‘fetal alcohol’ terminology in the absence of facial features raised concerns for some participants in the current study as it ascribes a level of causality to this group of individuals that is not present in diagnostic terms used in other international guidelines (i.e., neurobehavioural disorder, alcohol-exposed in The University of Washington 4-Digit Code [8]; alcohol-related neurodevelopmental disorder in the Revised IOM Guidelines [26]) that generate a spectrum of diagnoses when there are no facial features that describe alcohol as being related or associated with an individual’s outcome. However, the Canadian and Australian Guidelines suggest that the use of FASD as a diagnostic term simplifies the diagnostic process [1]. Given the inconsistencies in diagnostic terms, this is a topical and common area of debate that requires further consideration and exploration of opportunities to harmonise terminology internationally.

Relatedly, participants expressed a desire for greater clarity regarding the threshold for prenatal alcohol exposure, particularly in the context of using FASD as a diagnostic term. In the current Australian Guide, the diagnosis of FASD requires confirmed alcohol exposure in the absence of all three sentinel facial features (and unknown prenatal alcohol exposure in the presence of three sentinel facial features). Some international guidelines including the Canadian [1], Revised IOM [26] and the proposed DSM criteria [23] include a recommended minimum threshold of prenatal alcohol exposure to make a diagnosis. Although the risk of adverse outcomes increases with higher amounts and frequency of alcohol exposure, there is currently no known safe level of alcohol use during pregnancy. This is largely due to a range of methodological limitations inherent in the research [30] along with a wide range of factors that can interact with alcohol exposure to influence prenatal development, including prenatal nutrition, stress, and maternal and child genetics [31,32]. As a result, the maximum maternal blood alcohol level achieved following alcohol intake and transferred to the fetus varies considerably between individuals. Additionally, in the clinical setting, determining a specific level of prenatal alcohol exposure can be difficult. Information regarding prenatal alcohol exposure is not routinely performed or collected, and when it is, the specific screening tools used are often not validated for use in pregnant women [33]. Furthermore, a high proportion of individuals with FASD reside in out-of-home care settings [34], and thus clinicians are reliant on birth and child protection records or retrospective accounts from family/close contacts to obtain this information [35]. Consequently, this is a challenging area to review and will require careful consideration of the available evidence, and the potential risks and benefits of different approaches.

#### 4.1.2. Guideline Content

Priorities specific to guideline content highlighted the desire for guidance on communicating the diagnosis to individuals and families, minimum standards for diagnostic reports, and recommendations for post-assessment management and support plans. Feedback regarding the diagnostic outcome is an important but challenging aspect of any diagnostic assessment for both individuals and families receiving the diagnosis and for health professionals communicating the diagnosis. For individuals and their caregivers, previous research found that receiving a diagnosis of FASD can be a positive and empowering process by improving understanding of an individual’s daily challenges and increasing confidence to provide and seek appropriate support and services [36,37,38]. However, the experience can also be overwhelming related to the long-term support that will be required and feelings of guilt, shame and blame [39]. Little research has focused on health professionals’ perceptions and confidence in communicating a diagnosis of FASD to families, although research in the general medical profession suggests that many practitioners find such interactions stressful and lack training on how best to deliver difficult outcomes [40]. Literature within specific disability fields including Autism, Downs Syndrome and Cerebral Palsy has proposed guidelines for good practice in disclosing diagnoses. Recommendations emphasise consistent messaging across health professionals, the use of positive language, allowing families to react emotionally, providing time for families to ask questions, as well as discussing and setting future goals for the individual and providing recommendations and contacts for future support [41,42].

Diagnostic reports play a central role in supporting the communication of the diagnosis by providing a framework to understand an individual’s strengths and areas of vulnerability and are a particularly important resource used by families post-diagnosis in advocating for and securing further support and services [37,43]. Providing minimum and universal reporting standards may promote consistency, transparency and best practice across diagnostic services and could ensure that the needs of individuals with FASD are understood and can be met across a broad range of services and supports including other health, education, child protection and justice settings. Detailing recommendations for future support is a key aspect of the diagnostic report, although research has found that some caregivers of children diagnosed with FASD can feel overwhelmed by the number of recommendations provided, not knowing where to start and perceiving the recommendations as beyond the reach of the family [37]. Further, Australian Aboriginal parents and caregivers have been found to engage with their child’s FASD reports and resources differently compared to non-Aboriginal parents and caregivers. They have also been found to conceptualise their children’s diagnosis and ongoing management differently [43]. Thus, considering individual caregiver and family background and circumstances is also important in the formulation of recommendations and support plans.

Cultural considerations were highlighted by participants as an important priority, including proposals that the guide should be culturally sensitive, safe, and inclusive. They specified the need to consider the cultural appropriateness of assessment tools, clinical decision-making processes and the support provided post-assessment. Many assessment tools have not been validated among First Nations peoples and therefore often fail to capture the true, holistic picture of a First Nations person. In addition, assessments completed by non-First Nations professionals with First Nations peoples involve a complex array of issues additional to differences in worldviews. To be First Nations in a colonised country, such as Australia, has implications of issues of land sovereignty, barriers to accessing resources, imbalanced power dimensions, and intergenerational trauma. To address the holistic health needs of First Nations peoples, the consideration of equity imbalances and the social determinations of health is vital [44,45]. Responses to FASD for First Nations people must be culturally specific to individuals in the context of their families and communities [46]. It also requires co-design and collaboration with the community [47], with a specific focus on decolonising approaches [48] in order to ensure ownership in the hands of the community [49]. Cultural safety must be embedded and underpin the assessment and diagnostic process, regardless of the cultural background of the individual being assessed [43,50]. The extent to which tools and assessment processes will require further knowledge translation will be determined by the context in which the individual is presenting. Culturally adapted resources developed in partnership with interpreters, cultural advisors and consumers will facilitate safe and sensitive communication and practices throughout the diagnostic assessment process for individuals and their families.

#### 4.1.3. Guideline Dissemination and Implementation Considerations

There was a desire among stakeholders for the dissemination of the revised guide beyond the health system to include education, justice, child protection and the general community. Previous surveys of health, education and youth justice professionals report a lack of knowledge and training among staff to confidently recognise and manage individuals with FASD [51,52,53,54]. This lack of professional knowledge and understanding of FASD is reflected in caregiver reports of ongoing challenges and frustrations regarding the continual need to initiate and lead conversations about FASD with doctors, mental health workers, teachers, and other professionals in order to see progress [43,55]. To better support individuals with FASD, there is a critical need to facilitate awareness, recognition and understanding of FASD within the wider professional and general community. This does not require that these individuals share the same depth of knowledge as those involved in the diagnosis. However, early meaningful engagement and consultation throughout the guideline development process with key stakeholders at this broader level will likely encourage successful dissemination and uptake beyond core diagnostic practitioners. Implementing professional training opportunities alongside the guide may also support uptake and engagement with recommendations following the assessment.

Implementation considerations are primarily related to the validity of the guidelines, with stakeholders describing priorities for the revised guide to include an up-to-date evidence-base whilst also considering end-user perceptions, needs and values, and alignment with international best practices. A rigorous and transparent guideline development process is important for establishing trustworthiness and facilitates the successful implementation of the resulting guideline recommendations [56,57]. Although historically, guideline development has primarily relied on expert opinion and consensus in the absence of high-quality empirical evidence [58], recent international best practice recommendations have emphasised the use of systematic and rigorous evidence review methodologies and stakeholder involvement that considers patient views and preferences to inform guideline recommendations [11,13]. These recommendations are also indicated in Australia’s National Health and Medical Research Council (NHMRC) Standards for Guideline Development [12], which is the nation’s peak body for developing health advice for the Australian community, health professionals and governments. For the NHMRC to endorse a revision of a specific set of clinical practice guidelines, these Standards for Guideline Development must be met. However, in the field of FASD, there are considerable challenges in establishing evidence-based diagnostic guidelines due to limitations in the research evidence that includes the accurate assessment of prenatal alcohol exposure, variability in outcomes associated with dose and timing of prenatal alcohol exposure and limited empirical evidence of diagnostic specificity and sensitivity. Not surprisingly, there is no international consensus on criteria for diagnosis of FASD and there is variation in diagnostic practices internationally reflected in nine diagnostic guidelines currently published in the literature.

### 4.2. Strengths and Limitations

A strength of the current study is the inclusion of key stakeholders across a broad range of clinical disciplines required for the diagnosis of FASD. This engagement is important to ensure clinical relevance and enhance dissemination and implementation into clinical practice. The inclusion of stakeholders from other specialist areas including justice, education and cultural expertise is also a strength in understanding how the diagnosis of FASD can influence the identification and support of individuals with FASD beyond the clinical context, and importantly, highlighting cultural considerations for the Guide. There are several limitations, however, that should be considered when interpreting the findings. The survey relied on convenience sampling, resulting in a small sample due to the total number of participants in the Advisory Groups. The small sample size may have imbedded selection bias and it is likely that healthcare practitioners who are routinely involved in the assessment and diagnosis of FASD were more likely to express an interest in participating and thus complete the online survey. Although feedback from such stakeholders is important for understanding priority improvements to clinical practice, engaging stakeholders who have less experience could have provided an opportunity to identify priority areas to increase uptake from other practitioners. Despite the high number of participants raising cultural considerations as important, the low number of individuals identifying as having cultural expertise was a limitation. There was also limited representation from individuals and representatives with lived experience compared to professionals, which may have skewed priorities toward those of healthcare practitioners. The limited response rate of participants with cultural expertise and lived experience may reflect online survey methods not being the most appropriate data collection approach. Further feedback on priorities for the revised Guide is planned to be sought from cultural and lived experience advisory group members through stakeholder meetings and individual interviews as required. Nevertheless, this study documents the experiences and preferences of individuals with FASD and their carers and families, which are vital for ensuring the revised Guide meets the priorities and needs of individuals with lived experience who will ultimately be most affected by the diagnosis [11,12,13].

## 5. Conclusions

National guidelines are required to promote consistent diagnostic practices for FASD in Australia and improve diagnostic capacity. The process of revision ensures an up-to-date guideline in an evolving field. Stakeholder involvement is key to ensure relevant guidelines and acceptance when it comes to implementation and uptake. Key stakeholder priorities for the review and update of the Australian Guide include considerations and revisions to the diagnostic criteria, particularly focused on assessment within and across neurodevelopmental domains, guideline content of communicating the diagnosis and standards for reporting, ensuring cultural sensitivity and inclusion, as well as considerations for dissemination and implementation.

## Figures and Tables

**Table 1:** Diagnostic criteria and categories for Fetal Alcohol Spectrum Disorder (FASD) according to The Australian Guide to the Diagnosis of FASD.

Diagnostic Criteria	FASD with 3 Sentinel Facial Features	FASD with <3 Sentinel Facial Features
***Prenatal alcohol exposure***	Confirmed or unknown	Confirmed
***Sentinel facial features***	Presence of all 3 of the following: - Palpebral fissure <3rd percentile - Smooth philtrum (rank 4 or 5 ^1^) - Thin upper lip (rank 4 or 5 ^1^)	Presence of all 0, 1 or 2 of the following: - Palpebral fissure <3rd percentile - Smooth philtrum (rank 4 or 5 ^1^) - Thin upper lip (rank 4 or 5 ^1^)
***Neurodevelopmental impairment***	Severe impairment (≤2 standard deviations or <3rd percentile) in at least 3 domains: - Brain structure/neurology - Motor skills - Cognition - Language - Academic achievement - Memory - Attention - Executive function, including impulse control and hyperactivity - Affect regulation - Adaptive behaviour, social skills, or social communication	

Note: Adapted with permission from Bower and Elliott, 2016 [7]; ^1^ Using University of Washington Lip-Philtrum Guides [8].

**Table 2:** Key stakeholder background demographics (*n* = 62).

Background Demographics	Frequency (%)
Gender	
Male	9 (14.5)
Female	53 (85.5)
Non-binary	0 (0)
Residing state/territory ^1^	
New South Wales	10 (16.4)
Victoria	8 (13.1)
Queensland	20 (32.8)
Western Australia	13 (21.3)
Tasmania	0 (0)
South Australia	3 (4.9)
Australian Capital Territory	0 (0)
Northern Territory	7 (11.5)
Advisory Capacity ^2^	
Clinician	41 (66.1)
Researcher	18 (29)
Cultural Expertise	2 (3.2)
Lived experience/consumer representative	7 (11.3)
Primary professional discipline ^3^	
Psychology/neuropsychology	16 (29.1)
Allied health (speech pathology, occupational therapy, physiotherapy)	9 (16.4)
Perinatal, paediatric and/or public health research	9 (16.4)
Paediatrics	7 (12.7)
Criminal/youth justice	5 (9)
Other	9 (16.4)
Years of experience in primary discipline, M (SD), range	17.05 (9.87), 2–44
Years of experience working with individuals with FASD, M (SD), range	9.84 (7.78), 0–30

^1^*n* = 61; ^2^ multiple group membership allowable; ^3^ *n* = 55 professional stakeholders.

**Table 3:** Key stakeholder priorities for the review of the Australian Guide to the Diagnosis of FASD.

Priorities	Frequency (%)	Example Participant Quotes
**Diagnostic criteria**	**82 (30.7)**	
***Neurodevelopmental criteria***	***56 (68.3)***	
Conceptualisation of domains	21 (37.5)	“Acknowledge the overlap of symptoms and that impairment in three of the 10 domains may not reflect widespread brain injury…The guideline needs to urge the use of clinical judgment in such situations.”
Definitions of impairment	7 (12.5)	“I wonder if the use of cut-off scores for FASD diagnostic determinations is appropriate and should be reviewed. Some individuals can score above -2SD and have significant functional impairment.”
Inclusion of functional assessments	5 (8.9)	“Direct functional assessment is not currently required when considering a FASD diagnosis. Informant reports might be provided, which can give some insight into functioning, and inform the adaptive functioning/social communication domain. However, many difficulties and the impact of them can be invisible, even to people within the direct circle of care…”
Review assessment tools and approaches	3 (5.4)	“Update example tests under each domain. Including indirect measures. Update of Considerations for each area.”
Inclusion of sensory processing	3 (5.4)	“Inclusion of sensory processing in the neurodevelopmental domains for assessment. Sensory processing is important for development in motor, attention, executive functioning, affect and adaptive behaviours as a self-regulatory factor but could be unrecognised as a major contributor to impairments.”
Review inclusion of academic achievement	2 (3.6)	“Academic achievement domain—if a person’s language and cognitive are severe, then their academics are also going to be severely affected—should this be a stand-alone domain?”
Review inclusion/conceptualisation of affect regulation domain	6 (10.7)	“Consideration/justification and evidence in including affect regulation in the diagnostic criteria.”
Consider separation of adaptive and social communication/skills	3 (5.4)	“I’m unsure if adaptive functioning and social communication should be the one domain.”
Alignment with other neurodevelopmental condition standards/guidelines	4 (7.1)	“Referencing other diagnostic guidelines such as Developmental Language Disorder under Language, and Developmental Coordination Disorder under Motor for consideration within domain rankings may be useful.”
Individual recommendations	2 (3.6)	“Re-labelling “cognition” as intellectual functioning. Cognition is all thinking abilities; IQ is only one cognitive domain. Referring to IQ as cognition is misleading and leads to confusion.”
***Prenatal alcohol exposure***	***6 (7.3)***	
Review/clarify prenatal alcohol exposure criteria	6 (100)	“Specificity: ensuring that there is adequate guidance/guardrails for clinicians so that the diagnosis of FASD is only given when antenatal exposure to alcohol is very likely to be a primary cause of the identified impairments.”
***Sentinel facial features***	***2 (2.4)***	
Review facial features criteria	2 (2.4)	“Review of the assessment of facial features, selection of normative charts referred to across different ages and also for different ethnicities (including Aboriginal).”
***Defining FASD***	***8 (9.8)***	
Clarifying the definition of FASD	5 (62.5)	“Clarify if FASD is/will be intended to impute causal status to prenatal alcohol exposure (by way of title). Current Australian guide appears to require causality. But this varies in research and practice. To ensure nomenclature matches intention to convey accurate messages to empower others decision making for optimum outcomes + to avoid misdiagnosis and misnomers akin to this.”
Consideration of ‘the spectrum’ of FASD	3 (37.5)	“Exploring the diagnosis as a spectrum disorder, as opposed to only including the severe end of the spectrum of people (i.e., acknowledging people living with mild to moderate impairments).”
***Simplifying diagnosis***	***3 (3.7)***	
Simplifying assessment and diagnostic process	3 (100)	“To make the diagnosis more straight forward.”
***Other***	***7 (8.5)***	
Other individual diagnostic/assessment considerations	7 (100)	“Look at current diagnostic criteria for FASD and where it is falling short and what needs to be altered for better diagnostic clarity.”
**Guideline content**	**91 (34.1)**	
***Lifespan considerations***	***13 (14.3)***	
Increased consideration of adults	4 (30.8)	“Clearer guidelines for adult assessment.”
Consideration of how assessment is completed in young children/early detection	9 (69.2)	“Review the neurodevelopmental domains in relation to new research on features in young children under 6 years old.”
***Cultural considerations***	***18 (19.8)***	
Cultural sensitivity/ safety/inclusivity	9 (50)	“Inclusion of an individual’s cultural perspective/understanding of health and development. For First Nations peoples, this should involve a process of co-design to ensure the cultural safety of the Guide. Doing so will contribute to decolonising the Guide and the diagnostic methodology underpinning it.”
Assessment tools/clinical decision making	9 (50)	“Consider alternative assessment processes (and recommended assessment battery/tools) that are more culturally safe and appropriate for Aboriginal and Torres Strait Islander people.”
***Formulation/differential diagnosis/comorbid conditions***	***18 (19.8)***	
Formulation/differential diagnosis	10 (55.6)	“Expand on Section E: Formulating a diagnosis—points about excluding other causes or conditions and assessing potential influence of other exposures and events.”
Consideration of comorbid conditions	8 (44.4)	“Additional advice/reminders regarding the importance of screening for child maltreatment/trauma and sleep disorders during FASD diagnostic assessments.”
***Feedback/reports and post-assessment support***	***37 (40.6)***	
Process of providing feedback/diagnosis	2 (5.4)	“Include in the guidelines recommended protocols and processes to reporting and feeding back assessment results to individuals and families.”
Consistency and dissemination of reports	4 (10.8)	“That diagnosis reports be uniform across clinics in Australia and other diagnostic groups.”
Review management plans/supports and resources	9 (24.3)	“Provide more guidance on developing an effective management plan, with reference to evidence-based practice where possible.”
Increased support/coordination for individuals and families	17 (44.7)	“Ensure that all clients who receive a FASD diagnosis have available support services that are easy to access, free of cost, accurate and knowledgeable…”
Early intervention	3 (8.1)	“Early intervention where possible.”
Follow-up	2 (5.4)	“Follow up on children diagnosed to provide insight into better practices for managing FASD.”
***Ethical considerations***	***5 (5.5)***	
Potential implications of diagnosis and misdiagnosis	3 (60)	“Addition of a section on the common consequences of misdiagnosis and encouragement that clinicians consider these negative consequences when weighing up the accuracy of diagnosis, e.g., poorly targeted interventions, stigma, blame and shame for communities, disempowerment, reinforcing systemic racism, misuse by the legal system.”
Consent for referral/assessment	2 (40)	“Consent is not regulated. FASD is stigmatising diagnosis and warrants control of what constitutes informed consent...”
**Dissemination considerations**	**15 (5.6)**	
Widespread dissemination, including health, education, justice, child protection and the general community	12 (80)	“To disseminate this amongst both professional people and the community.”
Targeted dissemination to MD teams	2 (13.3)	“Dissemination of guidelines to most useful clinical groups—encouragement of multi-disciplinary teams.”
Specific strategy for primary health	1 (6.7)	“To get this onto health pathways, supported with education through established educational pathways –Royal Australian College of General Practitioners, Public Health Networks, etc.”
**Implementation considerations**	**63 (23.6)**	
***Validity***	***17 (27)***	
Consideration and presentation of up-to-date research evidence	8 (47.1)	“Update and revise based on recent research, particularly reviews and meta-analyses, where available.”
Consideration/harmonisation with international diagnostic approaches	6 (35.3)	“Consideration of harmonisation of available diagnostic guides/criteria internationally.” “Ensure it’s in line with best practice internationally.”
Individual recommendations	3 (17.6)	“The guide needs to include acknowledgement of the current significant limitations in the literature in this area, e.g., no clearly established dose-effect relationship between alcohol and impairments, no Aboriginal Australian norms for facial features, no established cognitive phenotype of FASD.”
***Applicability***	***6 (9.5)***	
Applicability	6 (100)	“Patient centred language, non-judgemental, provide better words and ways to express concerns, also centred on hope for the future and maximising outcomes for affected children.”
***Accommodation: User needs/values***	***7 (11.1)***	
Incorporation of lived experiences	4 (57.1)	“Involvement of people with FASD and their families.”
Individual recommendations	3 (42.9)	“Consulting with clinicians, families, sub-populations...to maximise acceptability and usefulness of revised guidelines in different settings.”
***Accommodation: Human resources***	***8 (12.7)***	
Consider alternatives to multi-disciplinary teams to expand access	4 (50)	“Consider alternatives/additions to multi-disciplinary team process, and collection of assessment information that can be completed via non-clinicians.”
Focus/review multi-disciplinary team approach	4 (50)	“Further highlighting the needs for multidisciplinary teams (and not single clinicians).”
***Accommodation: Professional***	***9 (14.3)***	
Recommendation regarding level of training required	3 (33.3)	“Minimum training requirements for any health practitioner (Registered Discipline or not) to be eligible to make the FASD diagnosis.”
Increased general awareness and training across contexts	6 (66.7)	“Training in FASD awareness for those working in the health, mental health, justice, and other relevant sectors. Aboriginal trainers should be used in Aboriginal organisations.”
***Implementation: Barriers/facilitators***	***8 (12.7)***	
Access to prenatal care information	2 (25)	“Sharing of information from antenatal to postnatal service providers.”
Pathways of care	2 (25)	“Pathways are developed for children who show atypical development where there has been known exposure to prenatal alcohol.”
Individual recommendations	4 (50)	“Resources to allow regional and rural clinicians to better assess as usually significant time constraints utilised.”
***Implementation: Tools***	***6 (9.5)***	
List of clinics/practitioners	2 (33.3)	“Forming a register of practitioners and clinics who can diagnose FASD.”
Individual recommendations	4 (66.7)	“Case examples where space permits.”
***Evaluation: Monitoring***	***2 (3.2)***	
Evaluation and monitoring	2 (100)	“Monitoring and evaluating implementation.”
**Other**	**6 (16)**	
Prevention	8 (50)	“Focus on need for prevention, i.e., engaging with women of childbearing years, their partners, opportunistic interventions, i.e., as part of consultation regarding sexual health, contraception, lifestyle, nutrition, etc.”
Screening	8 (50)	“Consider adding recommendations regarding screening.”

## Data Availability

The data presented in this study are available on request from the corresponding author. The data are not publicly available due to ethical considerations.

## Supplementary Materials

The following supporting information can be downloaded at: https://www.mdpi.com/article/10.3390/ijerph19105823/s1, File S1: Survey.
