# Supplementary material for: Key Stakeholder Priorities for the Review and Update of the Australian Guide to Diagnosis of Fetal Alcohol Spectrum Disorder: A Qualitative Descriptive Study
Source: Int J Environ Res Public Health. 2022 May 10;19(10):5823. doi: 10.3390/ijerph19105823 (PMC9140557; doi:10.3390/ijerph19105823)
Supplement: Supplementary file 1 [file ijerph-19-05823-s001.zip › ijerph-1667610-supplementary.pdf]

Thank you for agreeing to take part in an Advisory Group for the Review of the Australian FASD Assessment and Diagnostic Guideline.

We are collecting the information in this survey from Advisory Group members. The information in this survey will be collated and summarized and used to facilitate discussions during Advisory Group sessions. We are also planning to use the responses to these questions for research purposes.

The next couple of questions are to gather some background information about you.

Please indicate which Advisory Group/s you are a member of?	☐ Clinician<br>☐ Other Specialist<br>☐ Researcher<br>☐ Cultural Experience<br>☐ Lived Experience
Which state/territory do you reside in?	☐ New South Wales<br>☐ Victoria<br>☐ Queensland<br>☐ Western Australia<br>☐ Tasmania<br>☐ Australian Capital Territory<br>☐ Northern Territory<br>☐ South Australia
What is your gender?	☐ Male<br>☐ Female<br>☐ Non-binary
What is your primary discipline area and/or work role?	
How many years of experience do you have in this discipline area?	
How many years of experience do you have working in FASD?	

For the next questions, please list and describe up to five priorities that you think are important for the review of the Australian FASD Assessment and Diagnostic Guide.

Priority 1:

Priority 2:

Priority 3:

Priority 4:

Priority 5:
